# The Association between Childhood Environmental Exposures and the Subsequent Development of Crohn's Disease in the Western Cape, South Africa

**DOI:** 10.1371/journal.pone.0115492

**Published:** 2014-12-16

**Authors:** Abigail Basson, Rina Swart, Esme Jordaan, Mikateko Mazinu, Gillian Watermeyer

**Affiliations:** 1 Dietetics Department, University of the Western Cape, Bellville, Western Cape, South Africa; 2 Biostatistics Unit, Medical Research Council of South Africa, Parow, Western Cape, South Africa; 3 Statistics and Population Studies Department, University of the Western Cape, Bellville, Western Cape, South Africa; 4 Department of Gastroenterology, Groote Schuur Hospital, Cape Town, Western Cape, South Africa, and the Department of Medicine, University of Cape Town, Cape Town, Western Cape, South Africa; CWRU/UH Digestive Health Institute, United States of America

## Abstract

**Background:**

Environmental factors during childhood are thought to play a role in the aetiolgy of Crohn's Disease (CD). However the association between age at time of exposure and the subsequent development of CD in South Africa is unknown.

**Methods:**

A case control study of all consecutive CD patients seen at 2 large inflammatory bowel disease (IBD) referral centers in the Western Cape, South Africa between September 2011 and January 2013 was performed. Numerous environmental exposures during 3 age intervals; 0–5, 6–10 and 11–18 years were extracted using an investigator administered questionnaire. An agreement analysis was performed to determine the reliability of questionnaire data for all the relevant variables.

**Results:**

This study included 194 CD patients and 213 controls. On multiple logistic regression analysis, a number of childhood environmental exposures during the 3 age interval were significantly associated with the risk of developing CD. During the age interval 6–10 years, never having had consumed unpasteurized milk (OR = 5.84; 95% CI, 2.73–13.53) and never having a donkey, horse, sheep or cow on the property (OR = 2.48; 95% CI, 1.09–5.98) significantly increased the risk of developing future CD. During the age interval 11–18 years, an independent risk-association was identified for; never having consumed unpasteurized milk (OR = 2.60; 95% CI, 1.17–6.10) and second-hand cigarette smoke exposure (OR = 1.93; 95% CI, 1.13–3.35).

**Conclusion:**

This study demonstrates that both limited microbial exposures and exposure to second-hand cigarette smoke during childhood is associated with future development of CD.

## Introduction

The pathogenesis of Crohn's disease (CD) remains poorly understood, but is thought to reflect a complex interaction between genetic susceptibility, a defective immune system, the gastrointestinal microbiome and environmental factors. North America and Europe have historically reported the highest incidence and prevalence rates of CD in the world. During the latter part of the 20^th^ century however, as incidence rates have begun to stabilize in these nations, a dramatic rise in CD incidence has been observed within developing nations, particularly as they become increasingly industrialized [1], [2]. Industrialization fosters population wealth as well as improvements in living conditions, sanitation facilities and hygiene practices. Several theories have been proposed to explain the link between industrialization and the rising incidence of inflammatory bowel disease (IBD), but the ‘hygiene hypothesis’, suggesting that immunological balance is negatively affected by a limited exposure to microbes during childhood, remains one of the most widely accepted [3]. Second-hand cigarette smoke exposure during childhood has also been extensively researched, however the evidence remains inconclusive [4]–[6].

Over recent decades the majority of the South African population has experienced advancements in socioeconomic status, improved living conditions and overall changes in lifestyle habits. It is possible that these changes are responsible for the rising incidence of CD reported in the Western Cape [7], [8]. There is however limited data evaluating the association between environmental factors in childhood and future CD in our local setting.

The aim of this study was thus to investigate the association between childhood environmental exposures and the subsequent development of CD, with specific emphasis on the timing of exposure.

## Materials and Methods

### Design and Setting

This was a case control study of all consecutive CD patients seen between September 2011 and January 2013 during their normally scheduled appointments at the two largest public-sector hospitals in Cape Town; Groote Schuur Hospital (GSH) and Tygerberg Hospital (TBH). Approximately 90% of the 3.5 million people who reside in Cape Town rely on the public health care sector [9]. These individuals are largely economically disadvantaged and unable to afford private health care. As such GSH and TBH, as state tertiary referral centers, treat the majority of CD patients within the greater Cape Town area. Control subjects for this study were identified from the same populations giving rise to the CD cases.

### Study Participants

Clinical records were used to confirm CD diagnosis, defined according to the European Crohn's and Colitis Organization (ECCO) guidelines [10]. Patients with a prior diagnosis of tuberculosis were excluded in accordance with the algorithm proposed by Epstein et al. [11]. Healthy controls, not related to the CD cases, were recruited in 3 ways; (1) family and friends of patients admitted to the referral-based spinal injury rehabilitation wards, (2) orthopaedic outpatients seen during normally scheduled appointments with their orthopaedic specialist, and (3) the hospital porter and security personnel. The recruitment method was standardized by use of a predetermined script. Controls were excluded if they had a prior diagnosis of tuberculosis, IBD or other immune-mediated diseases, any gastrointestinal disorder (e.g., irritable bowel syndrome), or any family history of IBD.

### Data Collection

Following informed consent, data was collected via an interviewer-administrated questionnaire, consisting of predominantly multiple choice questions all of which included a ‘do not know’ option to reduce answer bias. The same questionnaire was used for the case and control groups. To standardize the questionnaire administration process, the interviewer had a predetermined list of allowed ‘definitions and explanations’ to clarify a question for a participant if needed. Using this questionnaire information pertaining to patient demographics, as well as multiple childhood environmental exposures was collected. To minimize recall bias, participants had the option of completing the questionnaire at home and returning it to the clinic if they felt consulting family members may improve the accuracy of some responses. One year after study completion a total of 40 (10%) randomly selected participants completed the interviewer administered questionnaire for a second time in order to measure the agreement between repeated data for the questionnaire using a kappa statistic. Only data pertaining to the 3 age intervals was extracted in this process. Again, participants had the option of completing the questionnaire at home if they felt consulting family members may help with the accuracy of some responses. Data has been made publicly available via Figshare at: http://dx.doi.org/10.6084/m9.figshare.1159053. Disease characteristics of the CD patients have been described in detail elsewhere [12]. The relevant data is available via the link: http://dx.doi.org/10.6084/m9.figshare.1041586.

### Ethics Statement

Ethical clearance was granted by the Senate Research Ethics Committee of the University of the Western Cape (Reg. no. 11/3/16), the Human Research Ethics Committee of the University of Cape Town (Ref. no. 122/2011) and the Provincial Department of Health. All participants gave written informed consent.

### Statistical Analysis

The demographic data for the cases and controls is presented as frequencies (percentages) for categorical data, and as medians and interquartile range (IQR) for numerical data. Multiple logistic regression models were conducted to assess environmental risk factors and their impact on CD. Risk factors that were significant for (P<0.05) for a specific age interval were included in the 3 final models (0–5 years, 6–10 years, 11–18 years); all were adjusted for age at study enrolment, gender and ethnicity. Odds ratios and 95% confidence intervals were reported to measure the effect size. Risk factors with a cell frequency below 10 for any of the four cells in the cross tabulations of the risk factor with CD were not included in the models. Exact logistic regression was used for modeling with small cell sizes. An agreement analysis was performed to determine the reliability of questionnaire data for all the relevant variables. The kappa statistic (ranging between 0 and 1, with 0 indicating no agreement and 1 indicating perfect agreement between the two occasions) was used to measure the agreement between repeated data for the questionnaire. Standards by Landis and Koch [13] were used to interpret the strength of the agreement.

## Results

Over an approximate seventeen month period, 194 CD patients and 213 controls meeting our inclusion criteria were identified. Eleven subjects (2 cases, 9 controls) that were approached refused to participate (response rate of 99% and 96%, respectively). Demographic and baseline characteristics for the case and control groups are shown in Table 1. Overall, 125 (31%) of the cohort were male and 281(69%) were female. There was a significant difference in the median age between the case and control group at study enrolment [47.0 (IQR 38.0–57.0) years and 32.0 (IQR 24.0–44.0) years, respectively, P<0.001]. Ninety seven percent of case and control subjects were born in South Africa (P = 0.02). The majority (99%) received a monthly income below R10, 000 (P = 0.39).

**Table 1:** Baseline characteristics of cases and controls at study enrolment.

	Cases (*n* = 194)	Controls (*n* = 213)	P value
Gender, no. (%)			
Male	53 (27)	72 (34)	0.20
Females	141 (73)	141 (66)	
Age at enrolment (median and IQR), yr.	47.0 (38.0, 57.0)	32.0 (24.0, 44.0)	<0.001
Age of CD onset (median and IQR), yr.	28 (21.5, 38.0)	NA	
Disease duration (median and IQR), yr.	16 (10.0, 24.0)	NA	
Married, no. (%)*	93 (48)	72 (34)	0.01
Ethnicity, no. (%)			
White	35 (18)	19 (9)	0.01
Coloured	152 (78)	177 (83)	
Black	7 (4)	17 (8)	
Born in south Africa, no. (%)			
Yes	184 (95)	211 (99)	0.02
No	10 (5)	2 (1)	
Occupation, no. (%)†			
Unemployed or housewife	92 (48)	57 (27)	<0.001
Farmer/laborer/domestic	61 (32)	94 (45)	
Office work, student	38 (20)	60 (28)	
Income per month, no. (%)			
R≤10,000	191 (98)	213 (100)	0.39
R>10,000	3 (2)	0 (0)	
Educated, no. (%)‡	36 (18)	38 (19)	<0.001
Smokers, no. (%)	104 (54)	105 (49)	0.27

NA; not applicable.

CD, Crohn's disease; IQR, interquartile range.

*Civil marriage or living with a partner.

† Missing data for 3 cases and 2 controls.

‡ At least some tertiary education.

### Environmental factors during the age intervals; 0–5 years, 6–10 years and 11–18 years

The results of the multiple logistic regression analysis evaluating environmental risk factor exposure in 3 age groups (0–5 years, 6–10 years and 11–18 years) are shown in Table 2. During the age interval 0–5 years; sharing bathroom with 3 or less people [(OR = 0.55; 95% CI, 0.31–0.97), (κ = 1.00; 95% CI, 1.00–1.00)] was protective against developing CD, whereas having piped tap water and bottled water as the primary source of drinking water [(OR = 2.10; 95% CI, 1.20–4.00), (κ = 0.63; 95% CI, 0.37–0.89)], second-hand cigarette smoke exposure [(OR = 1.71; 95% CI, 1.01–2.94), (κ = 0.75; 95% CI, 0.52–0.98)] and never having consumed raw beef [(OR = 2.84; 95% CI, 1.17–7.56), (κ = 0.60; 95% CI, 0.43–0.68)] were associated with CD development. An increased risk-association was also observed in subjects who never consumed unpasteurized milk [(OR = 8.02; 95% CI, 3.19–23.28), (κ = 0.62; 95% CI, 0.17–1.00)]. During the age interval 6–10 years; sharing a bathroom with 3 or less people [(OR = 0.51; 95% CI, 0.28–0.90), (κ = 0.58; 95% CI, 0.30–0.86)] was protective against developing CD, whereas having piped tap water and bottled water as the primary source of drinking water [(OR = 2.05; 95% CI, 1.10–4.10), (κ = 0.89; 95% CI, 0.69–1.00)], never having had a donkey, horse, cow or sheep living permanently on the property [(OR = 3.10; 95% CI, 1.42–7.21), (κ = 0.84; 95% CI, 0.12–1.00)], never having consumed unpasteurized milk [(OR = 6.43; 95% CI, 3.02–14.81), (κ = 0.79; 95% CI, 0.39–1.00)] and never having consumed raw beef [(OR = 2.31; 95% CI, 1.00–5.80), (κ = 0.78; 95% CI, 0.66–1.00)] were associated with CD development. During the age interval 11–18 years the risk of CD development increased in subjects who never had a donkey, horse, cow or sheep living permanently on the property [(OR = 4.31; 95% CI, 1.36–16.14), (κ = 1.00; 95% CI, 1.00–1.00)], in those with second-hand cigarette smoke exposure [(OR = 2.03; 95% CI, 1.20–3.48), (κ = 0.60; 95% CI, 0.53–0.86)], in those who had never consumed unpasteurized milk [(OR = 2.69; 95% CI, 1.23–6.17), (κ = 1.00; 95% CI, 1.00–1.00)] and in those who had never had a helminth infection [(OR = 1.90; 95% CI, 1.00–3.71), (κ = 0.63; 95% CI, 0.36–0.90)] (Table 2). An independent risk-association was identified for; never having consumed unpasteurized milk (OR = 5.84; 95% CI, 2.73–13.53) and never having a donkey, horse, sheep or cow on the property (OR = 2.48; 95% CI, 1.09–5.98) during the age interval 6–10 years, respectively, as well as during the age interval 11–18 years for; never having consumed unpasteurized milk (OR = 2.60; 95% CI, 1.17–6.10) and second-hand cigarette smoke exposure (OR = 1.93; 95% CI, 1.13–3.35).

**Table 2:** Environmental risk factors over three age intervals; 0–5 years, 6–10 years and 11–18 years.

			0–5 years			6–10 years			11–18 years
	Cases n (%)	Control n (%)	Adjusted OR (95% CI)*	Cases n (%)	Control n (%)	Adjusted OR (95% CI)*	Cases n (%)	Control n (%)	Adjusted OR (95% CI)*
Primary source of drinking water									
Piped or bottled water	162 (84)	172 (80)	2.10 (1.20, 4.00)	171 (88)	183 (86)	2.05 (1.10, 4.10)	94 (194)	194 (91)	1.92 (0.80, 4.60)
Outside tap/borehole/well/river/dam	31 (16)	42 (20)		23 (12)	31 (14)		6 (19)	19 (9)	
Hot piped tap water									
Access	59 (32)	85 (40)	1.18 (0.71, 2.00)	77 (40)	99 (46)	1.52 (0.92, 2.54)	112 (58)	129 (60)	1.52 (0.93, 2.51)
No access	128 (68)	127 (60)		116 (60)	114 (54)		82 (42)	85 (40)	
Community type									
Suburban or Urban	136 (76)	146 (72)	1.21 (0.71, 2.10)	147 (80)	156 (74)	1.58 (0.91, 2.76)	152 (80)	168 (80)	0.97 (0.55, 1.70)
Rural or farm or informal settlement	43 (24)	58 (28)		36 (20)	54 (26)		38 (20)	42 (20)	
Total number of people in household									
5 or less	77 (41)	123 (59)	0.68 (0.43, 1.09)	79 (41)	130 (62)	0.67 (0.42, 1.06)	100 (52)	129 (61)	1.05 (0.66, 1.69)
6 or more	111 (59)	86 (41)		113 (59)	81 (38)		94 (48)	82 (39)	
Number of people sharing a bathroom									
3 or less	31 (17)	59 (29)	0.55 (0.31, 0.97)	27 (15)	62 (30)	0.51 (0.28, 0.90)	36 (19)	65 (31)	0.65 (0.38, 1.11)
4 or more	151 (83)	147 (71)		159 (85)	146 (70)		157 (81)	147 (69)	
Number of bedrooms in home									
3 or more	82 (44)	110 (52)	0.87 (0.69, 1.11)	87 (46)	116 (55)	0.87 (0.69, 1.10)	110 (57)	131 (61)	0.91 (0.72, 1.14)
2 or les	104 (56)	101 (48)		102 (54)	94 (45)		83 (43)	83 (39)	
Type of toilet facility									
Flush (own family or shared)	154 (81)	187 (89)	1.35 (0.69, 2.63)	166 (86)	190 (90)	1.62 (0.78, 3.38)	180 (94)	203 (95)	1.76 (0.60, 5.08)
Bucket, pit latrine, no facility	36 (19)	24 (11)		26 (14)	21 (10)		11 (6)	10 (5)	
Household pets									
No	95 (51)	81 (39)	1.47 (0.92, 2.34)	91 (48)	96 (45)	1.13 (0.71, 1.79)	88 (46)	102 (48)	1.01 (0.64, 1.59)
Yes	93 (49)	126 (61)		100 (52)	116 (55)		103 (54)	112 (52)	
Donkey/horse/cow/sheep on property									
No	172 (90)	179 (85)	1.67 (0.83, 3.45)	178 (94)	179 (84)	3.10 (1.42, 7.21)	187 (97)	199 (93)	4.31 (1.36, 16.14)
Yes	19 (10)	32 (15)		12 (6)	34 (16)		5 (3)	15 (7)	
Cigarette smoke exposure									
Yes	145 (78)	150 (71)	1.71 (1.01, 2.94)	149 (78)	158 (74)	1.63 (0.95, 2.83)	155 (80)	149 (70)	2.03 (1.20, 3.48)
No	42 (22)	60 (29)		42 (22)	55 (26)		39 (20)	65 (30)	
Unpasteurized milk consumption									
Never	169 (96)	139 (78)	8.02 (3.19, 23.28)	169 (93)	149 (73)	6.43 (3.02, 14.81)	178 (93)	179 (85)	2.69 (1.23, 6.17)
Once per year or more	7 (4)	39 (22)		12 (7)	54 (27)		13 (7)	32 (15)	
Raw beef consumption									
Never	164 (95)	163 (86)	2.84 (1.17, 7.56)	171 (92)	173 (85)	2.31 (1.00, 5.80)	174 (92)	184 (86)	1.48 (0.69,3.29)
Once per year or more	9 (5)	27 (14)		11 (96)	29 (15)		15 (8)	29 (14)	
Helminth infection									
No	82 (52)	107 (56)	0.87 (0.53, 1.42)	104 (58)	125 (63)	0.85 (0.53, 1.37)	166 (88)	171 (81)	1.90 (1.00, 3.71)
Yes	77 (48)	84 (44)		74 (42)	75 (38)		23 (12)	40 (19)	
Treatment for helminths									
No	72 (48)	88 (47)	0.99 (0.60, 1.63)	98 (57)	105 (53)	1.17 (0.73, 1.89)	152 (82)	155 (74)	1.70 (0.95, 2.97)
Yes	79 (52)	98 (53)		74 (43)	95 (48)		33 (18)	55 (26)	

*OR odds ratio adjusted for age at study enrolment, ethnicity and gender, and 95% confidence interval. Subjects who responded ‘do not know’ were excluded from analysis.

No significant association for the increase or decrease in risk for developing CD during the 3 age intervals was found for; hot piped tap water, community type, total number of people in the household, number of bedrooms in the home, household pets, or having been treated for intestinal worms (Table 2). Furthermore, no significant association was identified for; being breastfed as an infant [(OR = 0.74; 95% CI, 0.39–1.41), (κ = 0.81; 95% CI, 0.71–0.83)], or attending daycare during the first 6 months of infancy [(OR = 0.61; 95% CI, 0.35–1.06), (κ = 0.76; 95% CI, 0.64–0.94)] (Table 3).

**Table 3:** Environmental exposures during infancy.

		Cases (*n* = 193)	Controls (*n* = 213)	Overall* (*n* = 406)	Adjusted OR (95% CI)†
Breastfed as an infant, no. (%)					
	Yes	125 (79)	167 (85)	292 (82)	0.74 (0.39, 1.41)
	No	34 (21)	29 (15)	63 (18)	
Day care attendance during first 6 month infancy, no. (%)					
	Yes	33 (18)	67 (34)	100 (26)	0.61 (0.35, 1.06)
	No	150 (82)	132 (66)	282 (74)	

*Subjects who responded ‘do not know’ were excluded from analysis.

† Adjusted for age at study enrolment, gender and ethnicity. The odds ratio in the above table was obtained from the logistic regression models, where the environment risk factors were modeled by case/control group.

## Discussion

Environment plays a crucial role in the pathogenesis of CD and is believed to be primarily responsible for the rapid rise in CD incidence rates worldwide. Outside of North America and Europe, childhood environmental exposures and the timing of exposure have not been adequately explored. Within the South African population in particular, only limited data is available [14].

This study included all consecutive state-sector adult CD patients within the Western Cape, South Africa seen over a seventeen month period. The majority of findings were consistent with the hygiene hypothesis, that limited microbial exposure due to a more ‘sterile’ environment, particularly early in life, increases the risk of CD development [3]. This hypothesis is supported by the increased risk of CD in subjects who did not consume unpasteurized milk during childhood, those who did not consume raw beef, as well as in those whose primary water source was bottled or tap water. In Cape Town, a large majority of households in urbanized areas continue to have a horse, donkey, cow or sheep living on the property, albeit this practice has become less common over time. Results from this study suggest that never having one or more of these animals living permanently on the property as an independent risk factor for CD. This has been show previously and it is likely that contact with farm animals would increase antigen exposure, again supporting the hypothesis [15], [16]. Poor socioeconomic status is associated with helminth infection. Helminth infection is believed to play an immunoregulatory role in that it is associated with up-regulating the Th2 response which opposes the pro-inflammatory Th1 response associated with immune-mediated diseases, such as CD. In line with findings from a recent South African study [14], subjects who were not exposed to helminths during the age interval 11–18 years had a 1.9-fold increased risk for developing CD. The findings from this study lend support to the hygiene hypothesis and highlight the emerging role of the microbiome in the pathogenesis of CD [17], [18].

Over recent years, computerized DNA sequencing technologies has revolutionized how we view the human microbiome [18], [19]. Microbial cells outnumber human cells by a factor of 10 and directly affect human gene transcription and the development of immune structures [20]. The way this microbiome is cultivated however, the resultant gene-gene interactions, as well as potential epigenetic changes in gene expression that result from microbial exposure during childhood, are infinite [18], [19]. Furthermore, the seemingly protective paradigm has been challenged as the outcome of some disturbed microbial compartments appears to depend on the type of virus or bacteria, the timing of exposure, and an individuals' genetic predisposition [18], [19]. It is possible that these principles may explain the small, yet protective effect against CD of sharing a bathroom with 3 or less other people, as this is at odds with the other findings. Previous studies have also reported lack of hot tap water access as a significant risk factor for CD development however the present study did not support this association [21], [22]. Conversely, given the fact that sharing a bathroom with 3 or less other people was not identified to be independently protective against CD, there may be other environmental exposures, as well as cultural practices, which were not investigated, but which contributed to this ‘protective’ effect. This may also explain the significant female predominance in the CD cohort, although gender differences in health utilization factors may also be contributing, but this warrants future investigation. Interestingly, in the Western Cape, autoimmune diseases, especially systemic lupus erythematosus, are also seen much more commonly in women [23], [24].

Earlier studies evaluating the CD risk-association with passive cigarette smoke exposure during childhood has yielded inconsistent results [4]–[6], [21], [22], [25]–[27]. It is possible that several factors may have influenced these findings; namely, variations in study quality, lack of ethnic uniformity of study populations, case and control selection methods, or a possible dose-response relationship in passive smoke exposure. The present data revealed an independent risk-association between passive smoke exposure with CD during the age intervals 0–5 and 11–18 years.

Retrospective studies are subject to recall bias that may influence the accuracy of self-reported environmental exposures. In an attempt to evaluate the degree in which our findings may have been influenced by recall bias, 10% (n = 40) of the participants completed the questionnaire for a second time. For the majority of findings the kappa statistic ranged between 0.60–0.99 which strongly supports the reliability of the data. Notably, the questions were not solely dependent on a ‘cognitive’ memory during that time period; even if a subject could not personally recall this more ‘factual’ information (e.g., number of people in the home, type of toilet facility), it may often be fairly accurately obtained with the help of other family members. The participant response rate in this study was relatively high. This may have been because case and control subjects felt they had sufficient time to participate, or alternatively were able to schedule a later study enrolment date as they frequented the hospital regularly due to either future scheduled appointments with their physician, or for the visitation of friends and family admitted to the hospital. In this study, the socioeconomic standing of participants at study enrolment was comparable as 99% had a household income of below R10, 000 per month, despite 18% of the cohort having at least some tertiary education. There were no Jewish study participants.

This study has several potential limitations. There may have been recall bias about childhood environmental exposures and living conditions given the fact that the mean age at study enrolment was 38 years (making the time period that the questions referred more than 20 years), and that the difference in age between the cases and controls at time of study enrolment was approximately 15 years. In addition, parental education and socioeconomic status during childhood was not evaluated and this may have influenced the results. Finally, the identification of CD patients was hospital-based, therefore not population-based; however most subjects in state practice attend the 2 hospitals from where patients were recruited and findings are likely generalizable.

## Conclusion

In conclusion, this study demonstrates that many of the environmental factors associated with CD relate predominantly to the hygiene hypothesis in that decreased exposure to bacterial antigens may increase the risk of future CD. However, describing the complex interaction between the human microbiome, modifying genes, the cumulative effect of microbial exposure and their putative causal role in disease development will be the next major step towards understanding immune-mediated disorders.
